# The Modified Stenver’s View for Cochlear Implants – What do the Surgeons Want to Know?

**DOI:** 10.5334/jbsr.2059

**Published:** 2020-07-03

**Authors:** Fiona McClenaghan, Robert Nash

**Affiliations:** 1 The Royal National Throat Nose and Ear Hospital, GB; 2 Great Ormond Street Hospital for Children, GB

**Keywords:** cochlear implant, hearing rehabilitation, modified Stenver’s radiograph, otorhinolaryngology

## Abstract

Despite developments in electrophysiological testing, imaging remains the standard method to determine cochlear implant positioning. Whilst cone beam computed tomography is optimal, modified Stenver radiographs are easier to perform and are therefore commonly used. With recent debate as to the need for routine imaging in uncomplicated cases, the radiologist is increasingly faced with cases of abnormal anatomy or surgical error.

The primary interest is the positioning of the electrode array within the cochlea. This includes evidence of tip roll over or kinking and depth of electrode insertion, as both are independent predictors of hearing outcomes and may necessitate revision surgery.

## Introduction

**Figure 1:**
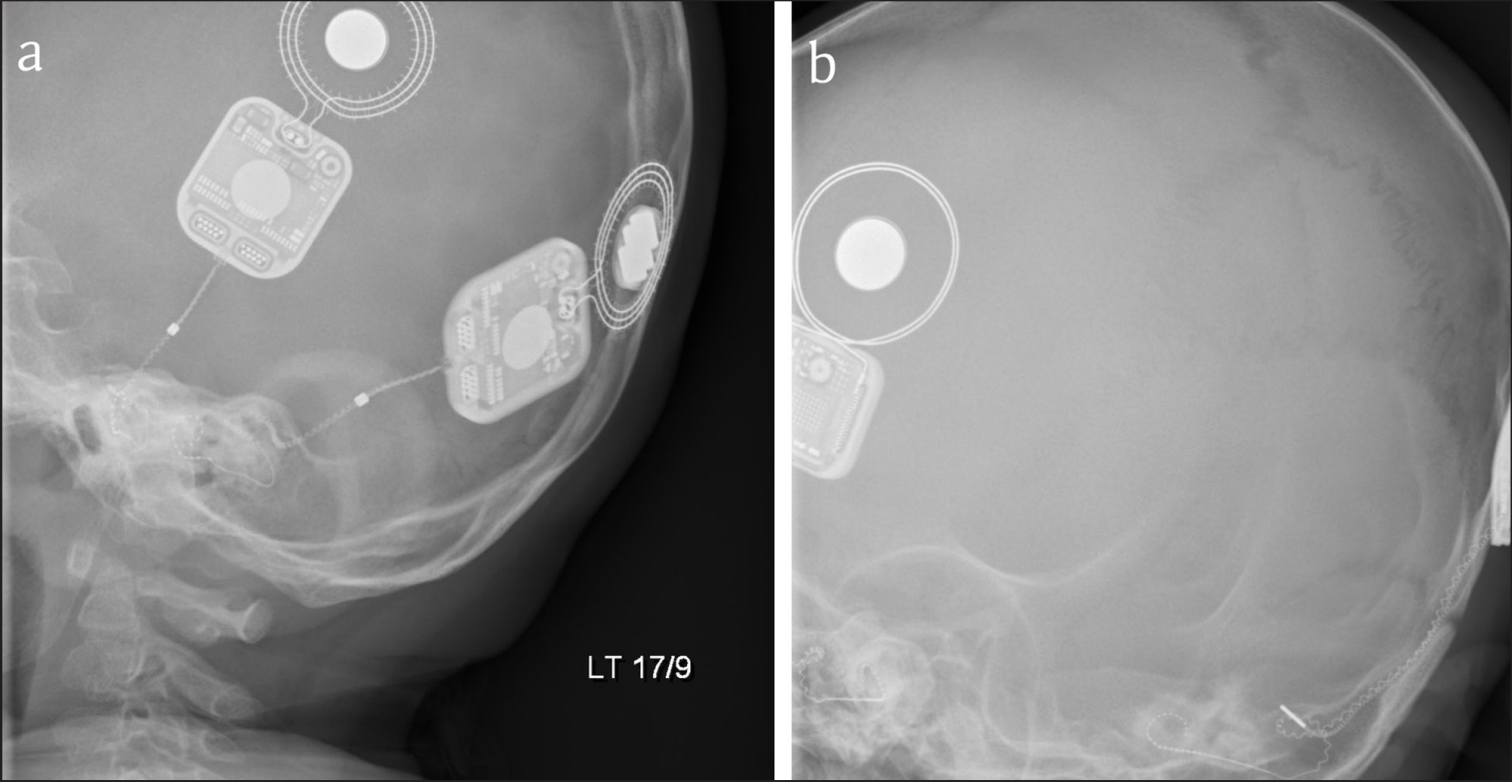
**a, b.** Stenver’s views of correctly placed bilateral cochlear implants with electrodes visible within the cochlea.

The modified Stenver’s view, first described by Marsh [1], has become the most commonly used radiological method to assess cochlear implant placement [2]. In the absence of international guidance regarding a preferred imaging modality, and indeed the need at all for radiological assessment of placement in patients with normal anatomy [3], what is required of the radiologist in reporting these radiographs can be unclear. Furthermore, radiologists who have not worked in cochlear implant centres may be unfamiliar with their use. The modified Stenver’s view in which the central beam through the temporal bone is at 45° posteriorly and 12° caudally demonstrates the petrous temporal bone, internal auditory meatus, and bony labyrinth. The oblique beam positions it in the plane of the superior semicircular canal and the electrodes can be visualised within the cochlea [1] (Figure 1).

## Discussion

**Figure 2:**
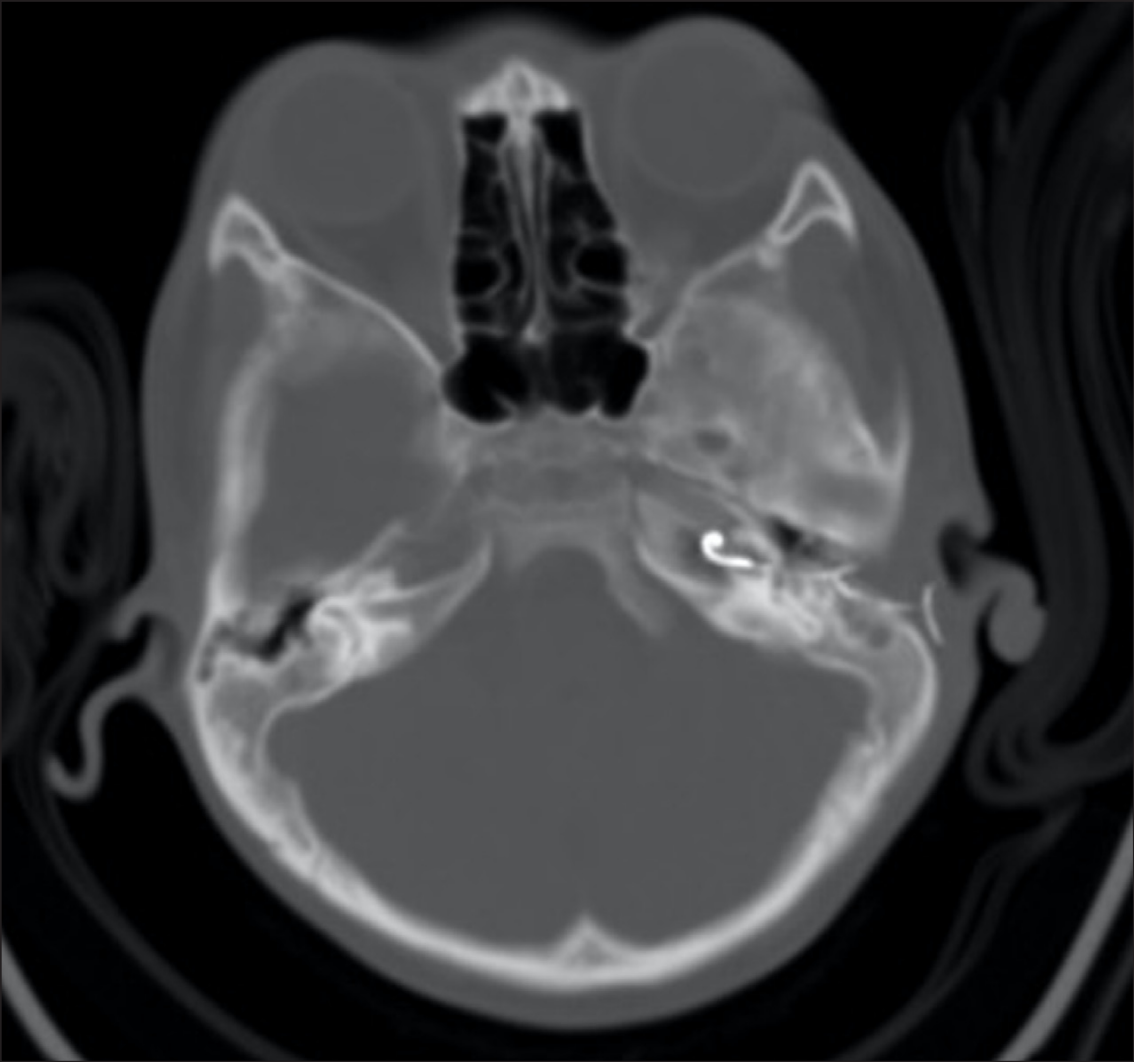
Axial CT image of a misplaced left cochlear implant electrode entering the carotid canal.

The correct placement of a cochlear implant is within the scala tympani. This provides optimum speech discrimination. It is exceptionally challenging to differentiate scala vestibuli and scala tympani on a Stenver’s radiograph, although computed tomography (CT) can be used for this purpose (particularly cone beam CT, or fusion with pre-operative MRI). Radiographic confirmation of intracochlear position and insertion depth is the primary aim of imaging and the gold standard for doing so [2]. Insertion depth can be estimated from the position of the ampullary limb of the semicircular canal, which is approximately in line with the conventional position for the cochleostomy/round window. Intraoperative electrophysiological testing can provide indirect assessment of electrode placement by measuring electrode impedance and electrically evoked compound action potentials, giving information on the integrity of the electrode and the neural responsiveness of the auditory nerve [4]; however, it cannot confirm correct positioning of the electrode array [5] and may be absent even with a correctly positioned electrode. Electrode array misplacements are rare but serious complications, often necessitating revision surgery with an incidence rate between 0.2–5.8% [2]. Reported sites for misplaced electrodes include the internal acoustic meatus, Eustachian tube, internal carotid artery, and superior semicircular canal [5,6] (Figure 2).

**Figure 3:**
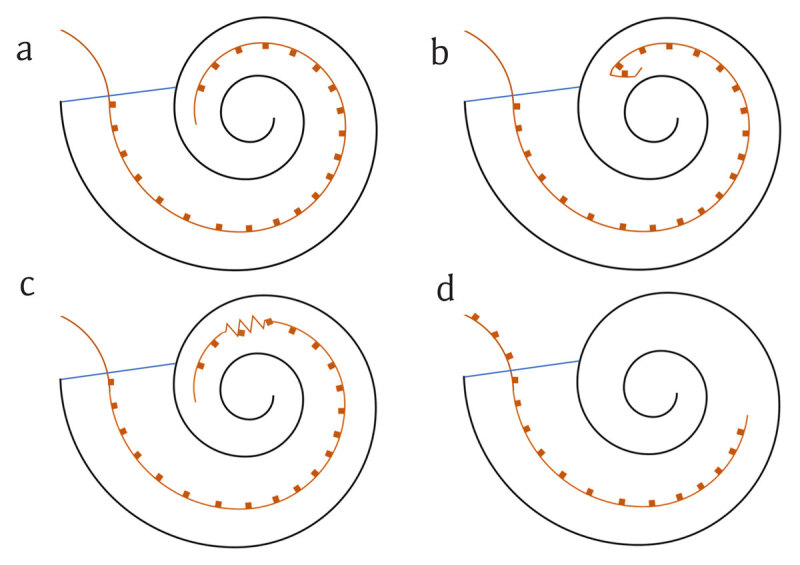
Schematic representation of intracochlear electrode placements. The orange line represents the electrode array and the orange squares represent active electrodes. The blue line marks the level of the cochleostomy (entrance to the cochlea). **a.** Full insertion into the cochlea. All active electrodes are medial to the cochleostomy. Optimal placement is when the most proximal active electrode is as close as possible to the cochleostomy but still within the cochlea. **b.** Electrode tip fold-over. The tip of the electrode has folded back on itself due to resistance to insertion while advancing the electrode. **c.** Electrode kinking. A portion of the electrode has twisted on itself due to resistance to insertion while advancing the electrode. **d.** Electrode under-insertion or extrusion. In under-insertion the electrode has not been advanced sufficiently so that all active electrodes are medial to the cochleostomy. Several active electrodes are therefore outside of the cochlea. In the case of extrusion, the electrode has slipped back via the cochleostomy, after insertion, leaving one or more electrodes lateral to the cochleostomy.

**Figure 4:**
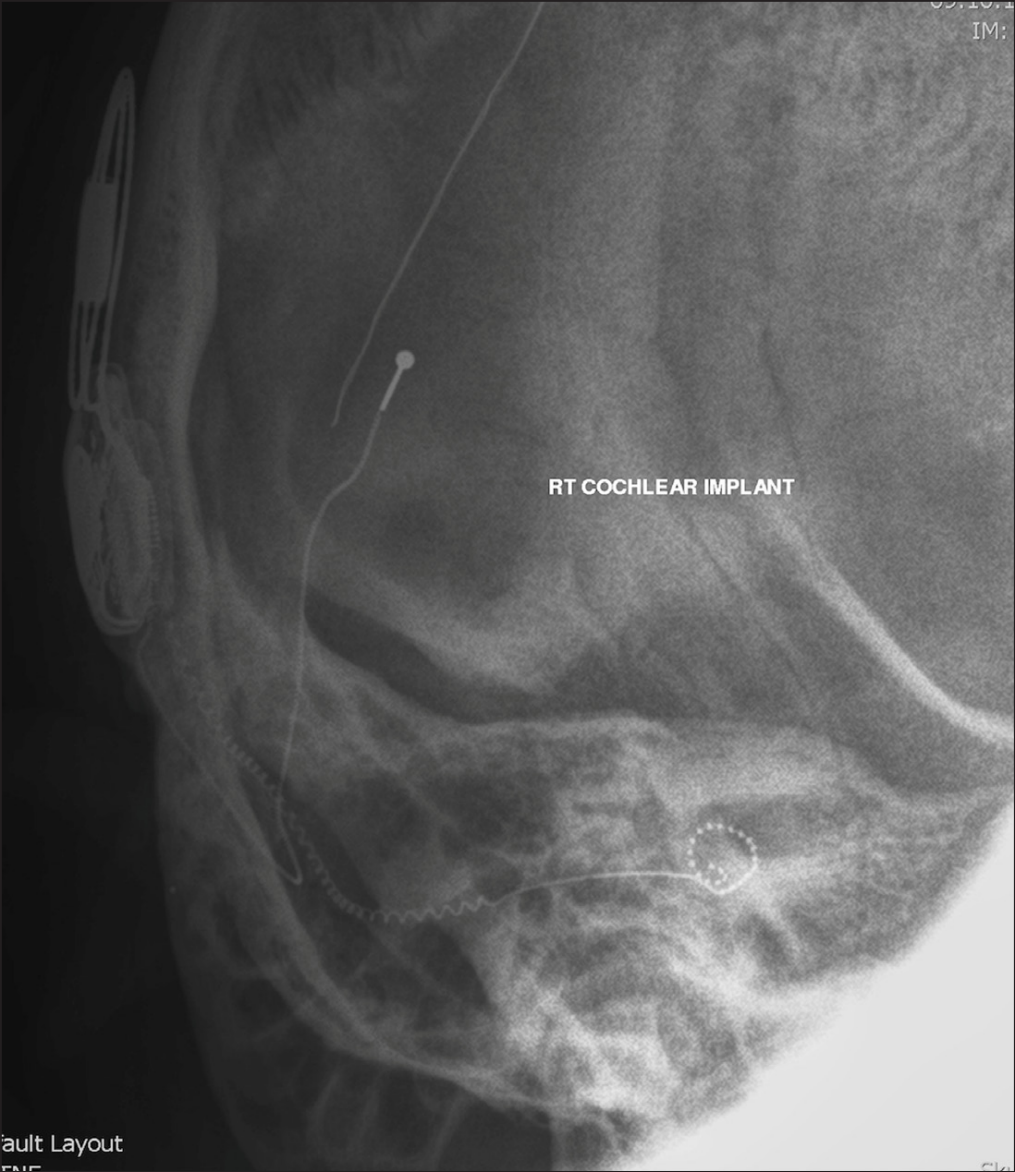
Modified Stenver’s view of a right cochlear implant with electrode tip roll over.

**Figure 5:**
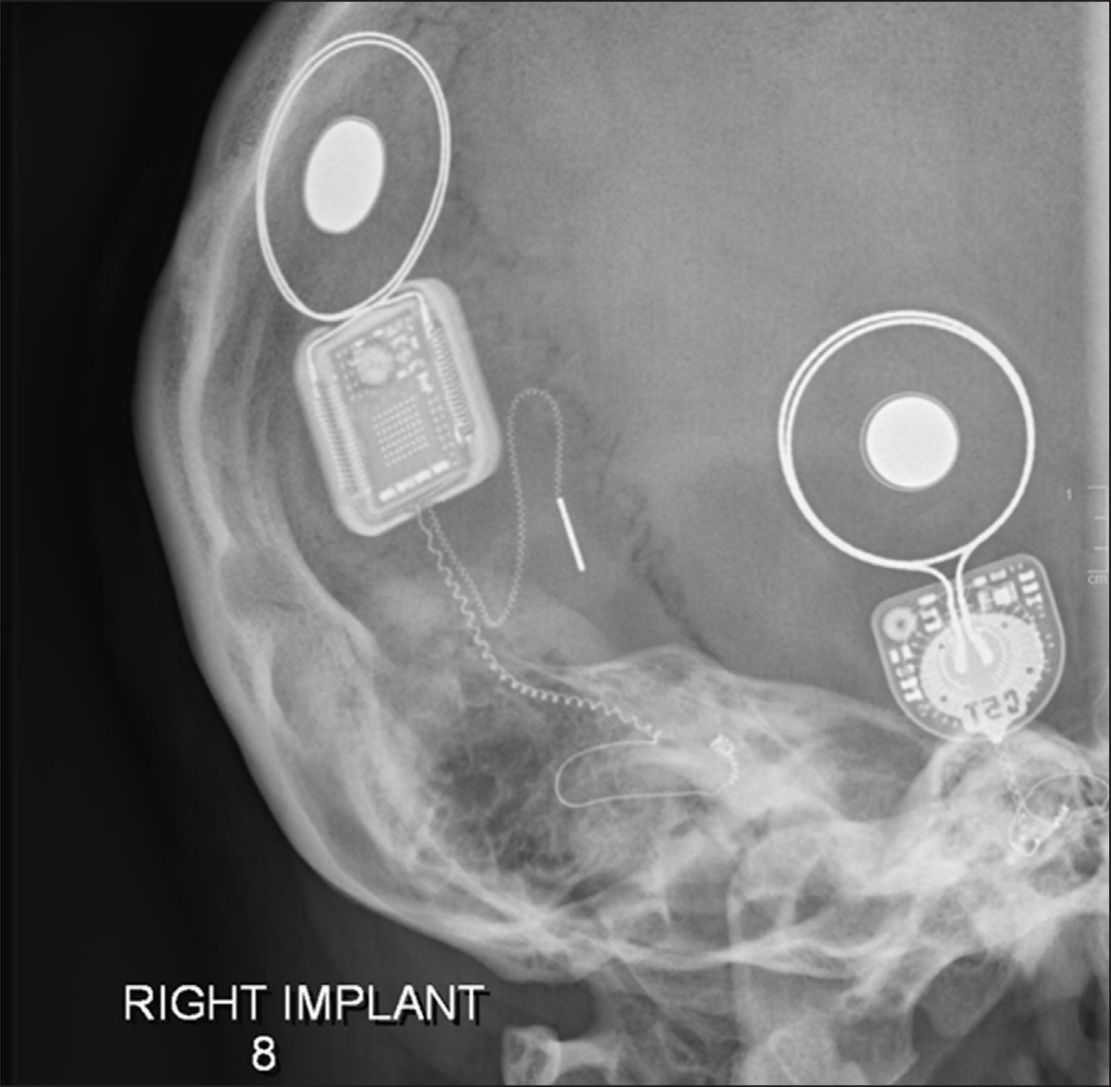
Modified Stenver’s view of right cochlear implant with electrode tip roll over and kinking leading to incomplete insertion.

Intracochlear misplacement is also seen, underinsertion, or extrusion and electrode tip fold-over or kinking [5] (Figures 3, 4 and 5). Electrode tip fold-over is the result of the electrode tip meeting resistance within the cochlea during insertion (Figure 4). If this is not noted, further advancement of the electrode can cause the tip to fold back on itself (Figure 3). This has been reported at a rate of 1.57% and electrode scalar deviation at 22.38% [7]. Characteristic intraoperative electrophysiological changes to predict tip fold-over have been described [5] but are often absent in radiologically confirmed cases [4,8]. Kinking also occurs when the electrode is advanced against resistance causing the electrode to curl upon itself and can occur at any point in the length of the electrode. The evidence regarding the impact of tip fold-over or kinking on clinical performance is limited, but such cases usually require revision, as the folded over or kinked segments affect current spread in the correctly placed electrodes.

**Figure 6:**
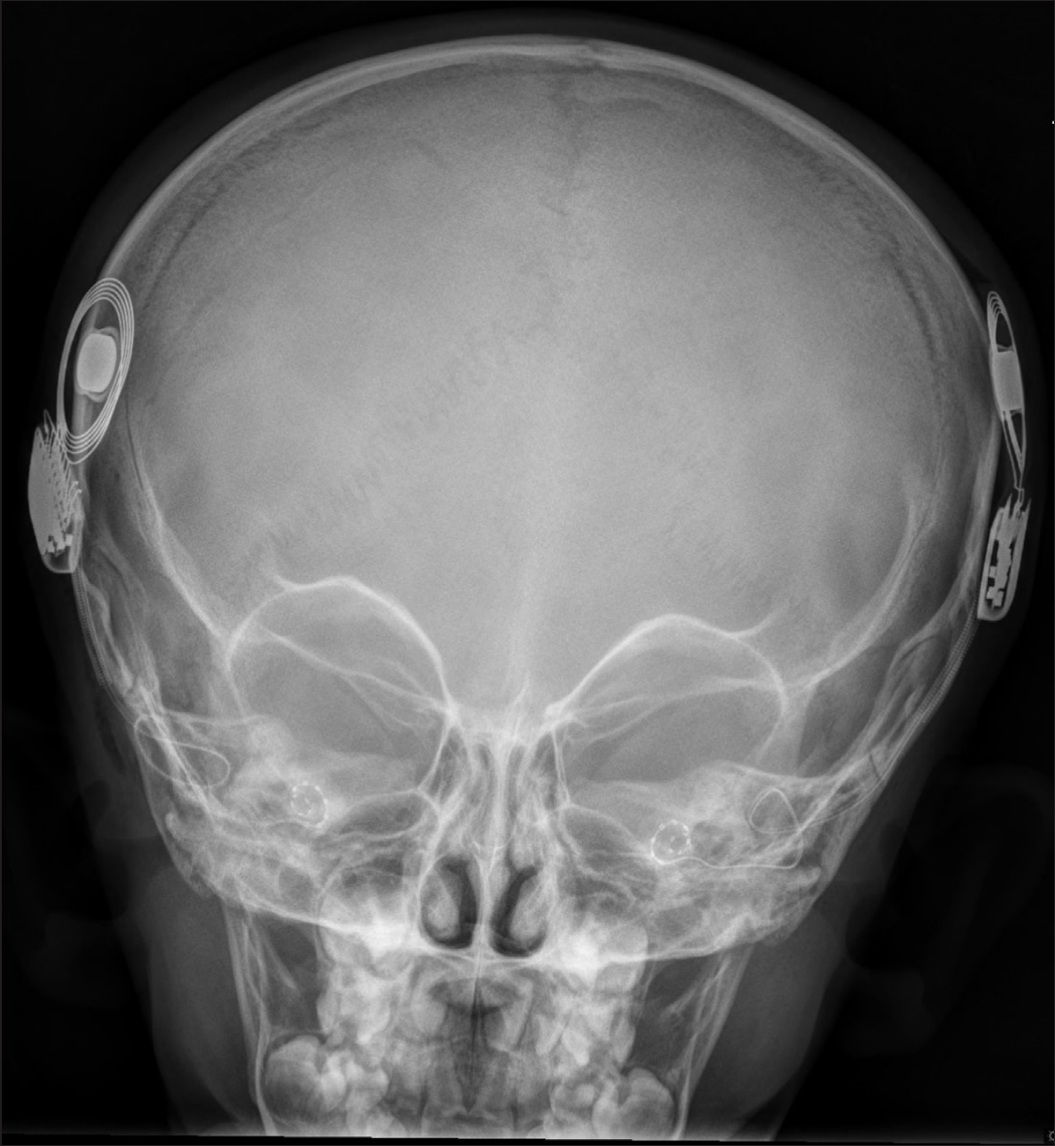
AP skull radiograph of correctly placed bilateral cochlear implants.

**Figure 7:**
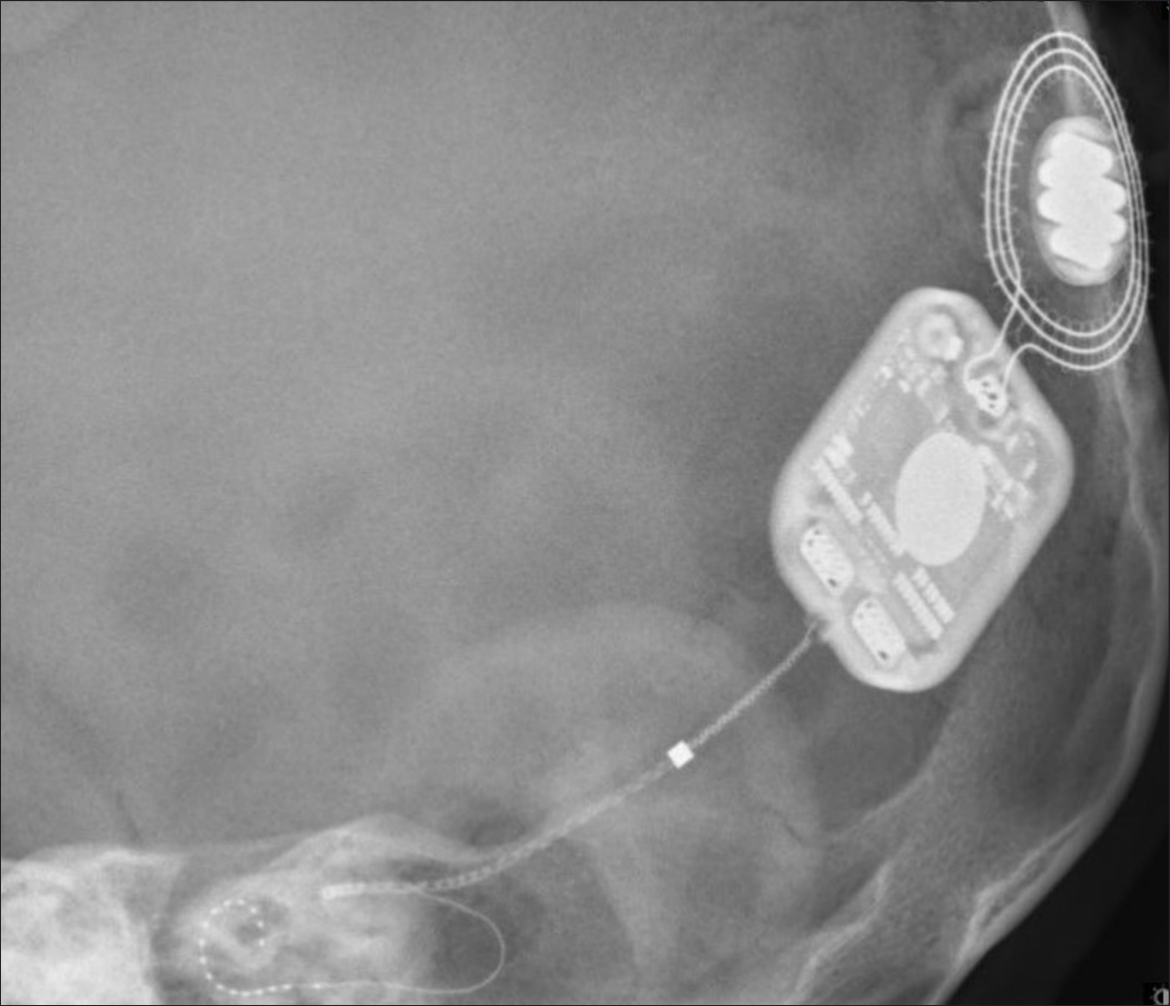
Modified Stenver’s view of a correctly placed left cochlear implant.

**Figure 8:**
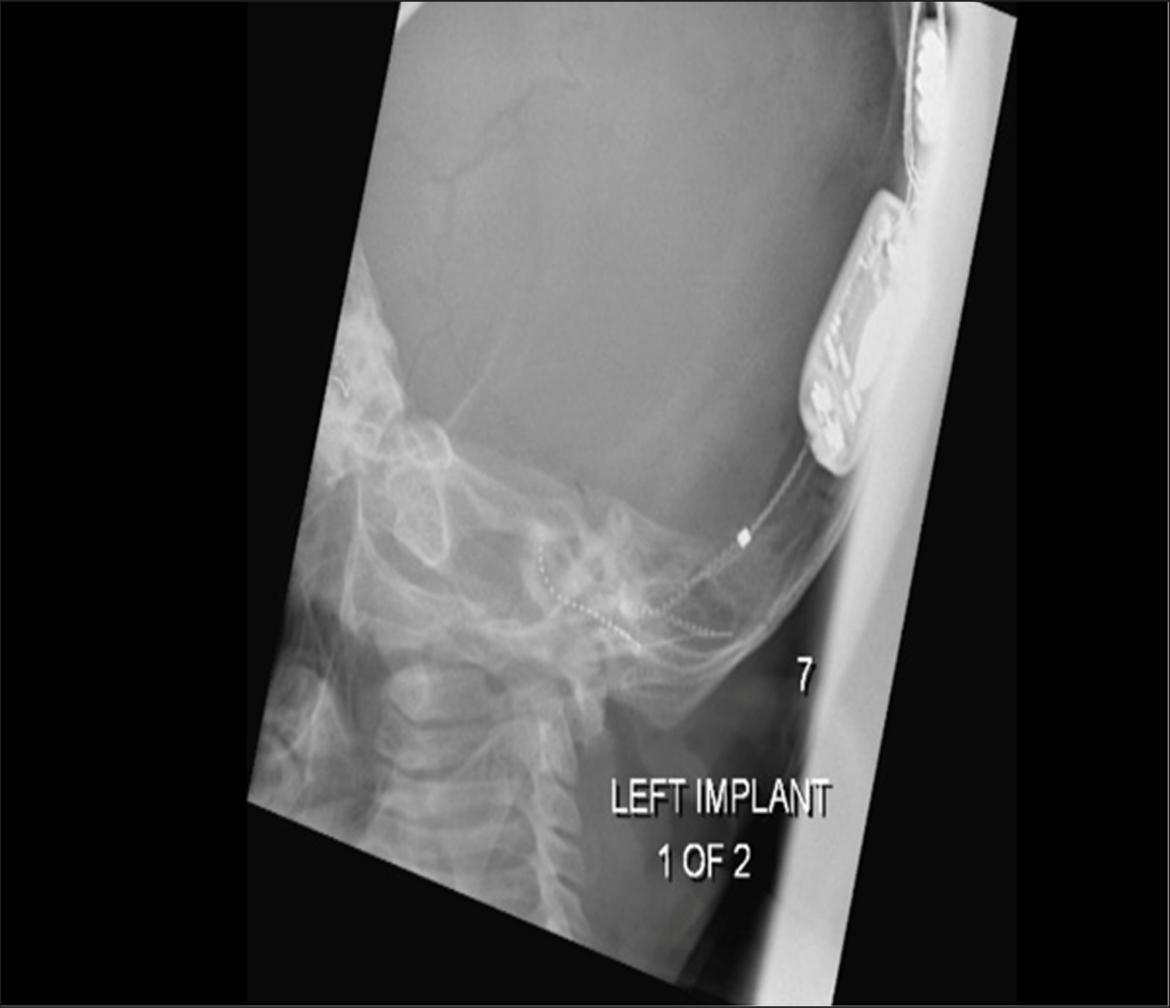
Modified Stenver’s view of an underinserted left cochlear implant.

**Figure 9:**
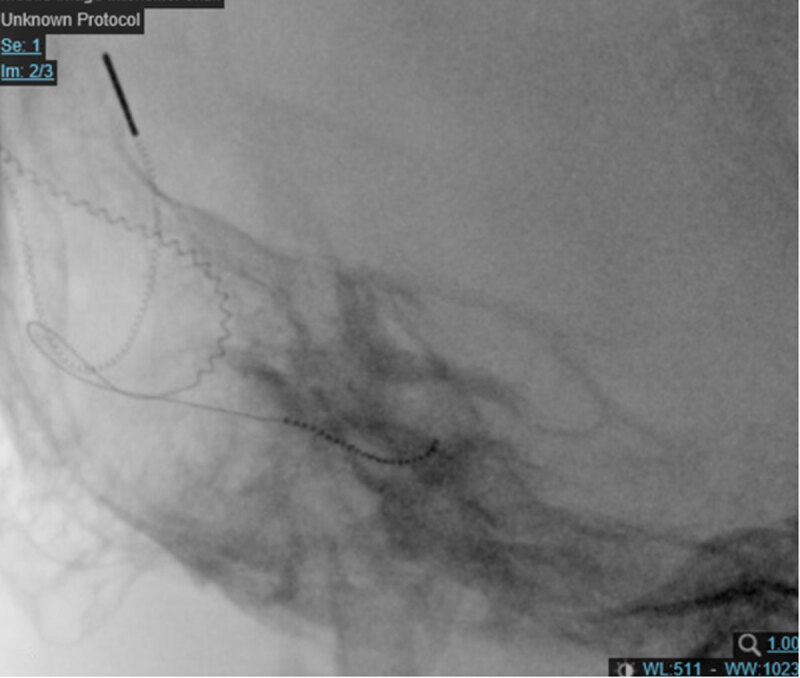
Modified Stenver’s view of an extruded right cochlear implant.

Depth of insertion of the array is important for hearing outcomes. Complete insertion of the electrodes is when all are medial to the line of the cochleostomy. This can be appreciated on a modified Stenver’s view as all electrodes lying within the cochlea (Figures 3, 6 and 7). Incomplete insertion is when one or more electrodes do not pass through the cochleostomy. This can be due to either incomplete insertion at the time of surgery or subsequent extrusion of the electrode from the cochlea. In both cases one or more electrodes lie outside of the cochlea (Figures 3, 8 and 9). Too deep or too shallow insertions correlate with poorer speech scores with deep insertions also correlating with poorer speech preservation [9]. When compared to electrophysiological testing and intra-operative counting of electrodes inserted, studies have reported variance between surgical estimates of insertion depth and radiological confirmation [1]. The modified Stenver’s view has excellent interrater variability in assessment of depth as well as position of electrode [4,10]. These radiographs also serve as a reference for future electrode migration should extrusion occur at a later date [2].

The routine use of plain radiographs to assess cochlear implants post-operatively has decreased due to concern regarding exposure to radiation, especially in the paediatric population [3], and the low incidence of electrode misplacement [2]. Some authors advocate the use of radiological confirmation only in cases of surgical suspicion of misplacement due to difficult insertion or abnormal patient anatomy [2,11,12]. However, while the specificity of surgical suspicion for misplacement is high at 88–99.5%, the sensitivity is only 55–60%. Therefore, relying on intra-operative suspicion alone to identify patients for radiological confirmation of implant location will miss a significant proportion of misplacements [2,13]. Imaging should also be considered in the patient whose auditory performance is persistently below expectations.

## Conclusion

Interpretation of the modified Stenver’s view after cochlear implantation remains a relevant skill for radiologists. Plain radiographs can reliably assess the position and depth of insertion of the electrode within the cochlea, and identify complications such as tip fold-over and extracochlear insertion. Accurate interpretation of such radiographs is essential to determine which patients require further imaging or revision surgery.
